# Epidemiological, clinical and laboratory characteristics of acute Q fever in an endemic area in Israel, 2006–2016

**DOI:** 10.1017/S0950268818003576

**Published:** 2019-03-01

**Authors:** S. Reisfeld, S. Hasadia Mhamed, M. Stein, M. Chowers

**Affiliations:** 1 Infectious Diseases Unit, Hillel Yaffe Medical Center, Hadera, Israel; 2 The Ruth & Bruce Rappaport Faculty of Medicine, Technion, Haifa, Israel; 3 Internal Medicine Department B, Hillel Yaffe Medical Center, Hadera, Israel; 4 Infectious Diseases Unit, Meir Medical Center, Kfar Saba, Israel; 5 Sackler Faculty of Medicine, Tel Aviv University, Ramat Aviv, Israel

**Keywords:** Acute, *Coxiella burnetii*, definitive diagnosis, Q fever

## Abstract

Our purpose was to describe the clinical, epidemiological and laboratory characteristics of patients hospitalised with acute Q fever in an endemic area of Israel. We conducted a historical cohort study of all patients hospitalised with a definite diagnosis of acute Q fever, and compared them to patients suspected to have acute Q fever, but diagnosis was ruled out. A total of 38 patients had a definitive diagnosis, 47% occurred during the autumn and winter seasons, only 18% lived in rural regions. Leucopaenia and thrombocytopaenia were uncommon (16% and 18%, respectively), but mild hepatitis was common (mean aspartate aminotransferase 76 U/l, mean alanine aminotransferase 81 U/l). We compared them with 74 patients in which acute Q fever was ruled out, and found that these parameters were not significantly different. Patients with acute Q fever had a shorter hospitalisation and they were treated more often with doxycycline than those without acute Q fever (6.4 *vs*. 14 days, *P* = 0.007, 71% *vs*. 38%, *P* = 0.001, respectively). In conclusion, acute Q fever can manifest as an unspecified febrile illness, with no seasonality. We suggest that in endemic areas, Q fever should be considered in the differential diagnosis in any febrile patient with risk factors for a persistent infection.

## Introduction

Q fever is a worldwide zoonotic infection, caused by *Coxiella burnetii.* The infection can be asymptomatic or manifest in a range from acute self-limiting disease to a persistent life-threatening infection. Diagnosis usually relies on serology, with specific immunoglobulins (IgM/IgG) [1].

Acute Q fever has a wide spectrum of clinical manifestations, including flu-like illness, pneumonia or hepatitis. The reporting of the clinical syndrome of acute Q fever differs globally; possibly due to host factors, inoculation dose, virulence factors and selection bias [1–4].

Q fever is endemic in Israel. The incidence ranges from 1 to 2 per 100 000 population [5] (and personal communication). The last data reported from Israel included 100 patients who were admitted to six medical centres from 1986 through 1996; many had hepatitis and thrombocytopaenia [6]. An epidemiological study by Bishara *et al*., in 2004, demonstrated an increased incidence among Jews, while no such increase was seen in the Arab population during the same period [5]. During the last 10 years, outbreaks were not reported in Israel (personal communication).

This study describes the clinical and laboratory features of patients admitted to our hospital and diagnosed with acute Q fever, to better understand the characteristics of the disease in an endemic area in Israel, in order to diagnose more accurately the patients that need close follow-up and treatment.

## Methods

Hillel Yaffe Medical Center is a 495-bed, university-affiliated, secondary hospital located in northern Israel. It serves a population of 450 000. This historical cohort study was conducted from 2006 through 2016. The study was approved by the hospital Institutional Review Board. Informed consent was not required.

Our hospital policy is to first screen serum for Q fever using the enzyme-linked immunoassay (EIA, ImmunoDOT™). This is a commercial qualitative assay that detects phase I and phase II antibodies, IgM or IgG and is considered positive for titres that are equivalent to immunofluorescent assays (IFA) titres of at least 1:10 for IgM both phases, 1:20 for phase II IgG and 1:1000 for phase I IgG. If positive, it is sent to the reference laboratory, and IFA is performed for confirmation and quantification. IFA is performed for phase I and phase II antibodies, IgM and IgG for each phase. A negative result is defined by the laboratory, when titre is lower than 100, and positive when titre is higher than 100 (100 is considered borderline), for both IgM and IgG. The decision to test for Q fever in each case is made by the treating physician, and is usually a part of work up for investigation of febrile diseases.

All patients, during the study period, that had IFA-positive samples, either for phase II IgG or IgM, were screened for the study. Positive results were defined as phase II IgM ⩾ 100, or phase II IgG ⩾ 200. Only the first serum in the study period was analysed for each patient. Additional inclusion criteria were age ⩾18 years and hospitalisations due to an acute illness compatible with acute Q fever (a flu-like illness, pneumonia, hepatitis in most of the cases and rarely pericarditis or meningo-encephalitis). Exclusion criteria were phase I IgG ⩾ 800, to avoid patients who might have had a persistent infection and cases with insufficient data.

Clinical, demographic, laboratory and imaging data were collected from patients' files, and data concerning repeated sera, if performed, were also analysed. Acute Q fever was definitively diagnosed when one of the following criteria was met: (1) phase II IgM ⩾ 50 and IgG ⩾ 200, (2) seroconversion of phase II IgG was demonstrated in repeated sera and (3) a fourfold increase in phase II IgG was demonstrated in repeated sera [7, 8]. These patients with a definitive diagnosis were compared to patients in whom diagnosis was definitely ruled out (definitely negative) (i.e. we only included patients that had paired sera taken at least 7 days apart, and did not fulfil diagnostic criteria, as mentioned above). Anaemia was defined if haemoglobin count was <12 g/dl, leucopaenia if white blood cell count was <4500 cells/μl and thrombocytopaenia if platelet count was <150 000/mcl. Pneumonia was defined when a new infiltrate was present in the chest X-ray, based on a radiology report. Rural origin was defined if it comprised 2000 inhabitants or less. Admissions between March and September were defined as spring and summer, while October to February was considered autumn and winter.

### Statistical analysis

Categorical variables were compared using the *χ*^2^ test or Fisher exact test, as appropriate. Continuous data were compared using the *t*-test or the Mann–Whitney *U* test, as appropriate. Multivariate logistic regression analysis was conducted including variables significantly associated with a definitive diagnosis of acute Q fever on univariate analysis (*P* < 0.05).

## Results

From 2006 through 2016, a total of 205 patients met the inclusion criteria, complete data were available for 152 patients, six patients had phase I IgG ⩾ 800 and were excluded from the study. Acute Q fever was definitely diagnosed, according to diagnostic criteria, only in 38 patients (30%). Although the diagnosis could not be ruled out, the remaining 87 patients did not meet the diagnostic criteria (mainly because repeat serum was not available). These 38 patients were compared to 74 patients from the same period of time, that were admitted to the hospital, and Q fever was ruled out with repeated serum. Demographic, clinical and laboratory data are presented in Table 1.

**Table 1.** Demographic, clinical and laboratory data of patients with and without a definitive diagnosis of acute Q fever, hospitalised during 2006–2016

Variables	Definitive diagnosis of acute Q fever, *n* = 38 (%)	Definitely negative for acute Q fever, *n* = 74 (%)	Odds ratio (95% confidence interval)
Age, years, mean (±s.d.)	51.1 (±17.3)	49.3 (±18.02)	1.01 (0.98–1.03)
Male sex	21 (55%)	37 (50%)	0.81 (0.37–1.78)
Arab ethnicity	3 (8%)	14 (19%)	0.36 (0.1–1.32)
Rural origin	7 (18%)	6 (8%)	0.4 (0.12–1.26)
Autumn or winter season	18 (47%)	26 (35%)	1.66 (0.75–3.68)
Any co-morbidities	20 (53%)	40 (54%)	0.94 (0.43–2.07)
Risk factors for chronic infection	2 (5%)	15 (20%)	0.22 (0.05–1.01)
Fever	37 (97%)	62 (84%)	6.57 (0.81–52.93)
Respiratory complaints	14 (37%)	34 (46%)	0.69 (0.31–1.53)
Headache	1 (3%)	18 (24%)	0.08 (0.01–0.66)
Rash	1 (3%)	4 (5%)	0.47 (0.05–4.39)
Creatinine, mg/dl, mean (±s.d.)	0.92 (±0.23)	1.1 (±1.5)	0.82 (0.48–1.38)
Anaemia	11 (29%)	32 (43%)	0.54 (0.23–1.24)
Leucopaenia	6 (16%)	11 (15%)	1.07 (0.36–3.17)
Thrombocytopaenia	7 (18%)	21 (28%)	0.57 (0.22–1.49)
Elevated liver enzymes	14 (37%)	7 (9%)	0.56 (0.23–1.36)
AST, mean (±s.d.)	76 (±249)	48 (±49)	1.001 (0.99–1.004)
ALT, mean (±s.d.)	81 (±250)	46 (±50)	1.002 (0.991–1.005)
CRP, mg/l, mean (±s.d.)	154.1 (±101.7)	113.6 (±104.6)	1.004 (1.00–1.01)
Pneumonia on X-ray	17 (45%)	21 (28%)	1.93 (0.85–4.37)
Treated with doxycycline	27 (71%)	28 (38%)	4.03 (1.73–9.38)
Length of hospitalisation	6.4 (±4.9)	14.2 (±17.6)	0.91 (0.84–0.98)

s.d., standard deviation.

Median age of the study group was 51 years (range 19–86), three (8%) were of Arab ethnicity, 31 (82%) lived in urban areas, 20 (53%) were admitted during spring and summer, and mean length of stay was 6.4 days. Haemoglobin level and platelet counts were within the normal range (mean 12.8 g/dl and 229 000/mcl respectively). Mean aspartate transaminase (AST), alanine aminotransferase (ALT), gamma-glutamyl-transpeptidase were elevated (76 U/l, 81 U/l and 100 U/l respectively), but alkaline phosphatase and bilirubin were within the normal range (mean 98 U/l and 0.8 mg/dl respectively).

More patients in the definitive group than in the suspected group had fever (97% *vs.* 84%), but less had headache (3% *vs.* 24%). C-reactive protein (CRP) tended to be higher in patients with acute Q fever compared to the control group (mean 154 mg/l *vs.* 113 mg/l, *P* = 0.068), but other laboratory findings were similar. Patients with acute Q fever had shorter hospitalisations and were more often treated with doxycycline.

## Discussion

In the 38 patients with a definitive diagnosis, fever was a prominent symptom, and CRP was slightly higher than in the control group. No imaging or other laboratory findings distinguished between patients in whom acute Q fever was suspected and confirmed and patients in whom acute Q fever was suspected and ruled out. Hepatocellular liver enzymes were slightly elevated in most of the patients but without a statistical significance between the groups, thus we assume acute Q fever was suspected in patients with fever and some laboratory abnormalities, especially elevated liver enzymes. We cannot generalise the results on all patients admitted with febrile illness in our area. In a previous study of acute Q fever in Israel, the clinical manifestations were somewhat different from those found in the current study; primarily hepatitis and thrombocytopaenia [6].

We believe the differences were mainly due to selection bias. Since our hospital is in an endemic area for both Q fever and spotted fever (boutonneuse fever), both tests are frequently requested by the treating physicians in febrile patients.

We did not find that ethnicity or rural inhabitance were risk factors for acute Q fever in our area, although these were described as risk factors in previous studies from other countries [9, 10]. Bishara *et al*. showed a lower incidence of Q fever in the Arab population compared to the Jewish population in Israel between 1991 and 2001 [5]. A possible explanation is that in Israel, house pets are a major reservoir of Q fever and not farm animals, as suggested by a well-described outbreak in the city of Tel Aviv, that probably originated in cats [11]. Housecats are more prevalent in the Jewish population than among the Arab population. This, of course, is a speculation, since we did not have accurate data about animal exposure or any data about the prevalence of Q fever in cats in Israel.

Our results indicate that acute Q fever was diagnosed throughout the year. Seasonal patterns have been described in endemic areas of Q fever, and are related to lambing season and wind, and in tropical areas to heavy raining [12]. In France and Spain for example, spring and summer were reported as risk factors for Q fever infection [9, 10]. The lambing season in Israel is throughout the year (synchronised by farmers), and if Q fever is linked to pets as well, its year-round appearance is not surprising.

Acute Q fever is a self-limited disease in many cases, and even when clinical treatment is recommended, it resolves without adverse sequelae in most patients [1]. However, among patients with risk factors, such as valvular disease, vascular aneurysm or grafts and malignancies, the risk for a persistent infection is substantial [13]. This risk is reduced dramatically with a prolonged antimicrobial treatment [14, 15]. Based on the data reported here, and to diagnose those at risk for complications, we suggest that in endemic areas, such as Israel, patients with risk factors for a persistent infection, and any febrile disease, should be tested for Q fever, even in the absence of other clinical or laboratory findings that usually lead clinicians to request this test.

This study has limitations. First, it is a small cohort. This is not unique to our medical centre, because in many cases after patients recover, the primary physician does not request repeat serum testing. There are variations in different immunoglobulins for the two phases of Q fever during 48 weeks after an acute infection. Phase II IgG may be elevated months after the acute infection, thus this marker by itself is not diagnostic of acute illness, and most laboratories require paired sera for a definitive diagnosis of an acute disease [7, 8, 16]. Since in many cases paired sera are unavailable, definitive acute Q diagnoses were uncommon and we had a small cohort for analysis. We believe our patients represent the population in Israel, because the hospital serves a population of 450 000, from both rural and urban areas, and of diverse ethnicities. The second limitation is that the study was conducted in Israel, and the results may not be generalisable worldwide. Many similar published reports, like ours, that focused on the acute form of Q fever, encountered similar selection bias, which could be a primary reason for the varying clinical manifestations of the disease worldwide.

In conclusion, this study presents updated information on patients with acute Q fever, from an endemic area in Israel. Clinical symptoms, laboratory results or imaging are nonspecific. A high index of suspicion is required for diagnosis and is especially warranted in patients at high-risk for progression to a persistent infection.
